# Dioxin Revisited: Developments Since the 1997 IARC Classification of Dioxin as a Human Carcinogen

**DOI:** 10.1289/ehp.7219

**Published:** 2004-06-10

**Authors:** Kyle Steenland, Pier Bertazzi, Andrea Baccarelli, Manolis Kogevinas

**Affiliations:** ^1^Rollins School of Public Health, Emory University, Atlanta, Georgia, USA; ^2^Department of Occupational and Environmental Health, EPOCA Research Center for Occupational, Clinical, and Environmental Epidemiology, University of Milan, Milan, Italy; ^3^Respiratory and Environmental Health Research Unit, Municipal Institute of Medical Research, Barcelona, Spain

**Keywords:** carcinogen, dioxin, TCDD

## Abstract

In 1997 the International Agency for Research on Cancer (IARC) classified 2,3,7,8-tetra-chlorodibenzo-*p*-dioxin (TCDD; the most potent dioxin congener) as a group 1 carcinogen based on limited evidence in humans, sufficient evidence in experimental animals, and extensive mechanistic information indicating that TCDD acts through a mechanism involving the aryl hydrocarbon receptor (AhR), which is present in both humans and animals. The judgment of limited evidence in humans was based primarily on an elevation of all cancers combined in four industrial cohorts. The group 1 classification has been somewhat controversial and has been challenged in the literature in recent years. In this article we review the epidemiologic and mechanistic evidence that has emerged since 1997. New epidemiologic evidence consists primarily of positive exposure–response analyses in several of the industrial cohorts, as well as evidence of excesses of several specific cancers in the Seveso accident cohort. There are also new data regarding how the AhR functions in mediating the carcinogenic response to TCDD. The new evidence generally supports the 1997 IARC classification.

## The 1997 IARC Evaluation

In 1997 the International Agency for Research on Cancer (IARC) classified TCDD (2,3,7,8-tetrachlorodibenzo-*p*-dioxin, the most potent dioxin congener) as a group 1 carcinogen (IARC 1997) based on limited evidence in humans, sufficient evidence in animals, and extensive mechanistic information indicating that TCDD acts through a mechanism involving the aryl hydrocarbon receptor (AhR), which is present in both humans and animals. The 1997 IARC evaluation updated an older, obsolete evaluation that had classified TCDD as a group 2B (possible) human carcinogen. IARC’s criteria for “limited” evidence for the epidemiologic studies requires that a causal interpretation is “credible,” but that chance, bias, or confounding cannot be ruled out as the source of the observed association. TCDD was unprecedented in that it was judged to cause an increase in cancers at all sites rather than at a few specific sites. This judgment was supported by both the epidemiologic and animal data. In animals, there were no “hallmark” sites that were elevated; instead, different tumor sites were elevated in different species in different studies. Furthermore, tissue concentrations were similar both in heavily exposed human populations, in which increased overall cancer risk was observed, and in rats exposed to carcinogenic dosage regimens in bioassays. At the 1997 IARC meeting held 4–11 February in Lyon, France, there was consensus that the epidemiologic evidence was at least “limited,” with some consideration that it was “sufficient.” The main discussion and division of opinions concerned the use of mechanistic data to interpret cancer risk in humans.

In 1997, the epidemiologic evidence consisted of studies of *a*) several industrial cohorts of chemical workers producing chlorophenol and phenoxy herbicides; *b*) cohorts of civilian or military pesticide applicators; *c*) the Seveso accident cohort; and *d*) numerous community-based studies. The IARC working group on dioxins summarized all of the available data but based the epidemiologic evaluation on studies of four highly exposed subcohorts within the industrial cohorts, and on the Seveso cohort. The main criteria for relying on these studies were principally that the cohorts included subjects with levels clearly higher than background and that exposure was well characterized. The four industrial cohorts are listed in Table 1, which also defines the subcohorts and their all-cancer mortality in reference to external populations. Exposure information is also given in Table 1 in terms of parts per trillion in serum. To put this in context, the general population has serum levels of approximately ≤ 5 ppt, and levels have been gradually decreasing in recent decades (Aylward and Hays 2002; IARC 1997; Schecter et al. 2003). The four industrial subcohorts were consistent in showing significant although moderate elevations of cancer mortality. When the data were combined, the standardized mortality ratio for all four subcohorts was 1.40 [95% confidence interval (CI), 1.1–1.7]. An exposure–response analysis was available in 1997 for two of the four cohorts (Flesch-Janys et al. 1995; Ott and Zober 1996); both of these analyses showed a significant positive exposure response for all cancers. Confounding by smoking or by other chemicals was judged to be unlikely to explain the observed consistent all-cancer excess.

## Evidence Published after 1997

### New exposure–response analyses.

Since the IARC monograph on dioxins (IARC 1997), there have been several new exposure–response analyses using the industrial cohorts (Table 1). These analyses have used similar techniques to develop estimates of serum TCDD levels for all workers in the cohort.

Using a newly developed job-exposure matrix (JEM) (Piacitelli et al. 2000), Steenland et al. (1999) analyzed exposure–response analysis in the NIOSH (National Institute for Occupational Safety and Health) cohort using cumulative exposure scores. The JEM was based on knowledge of the amount of TCDD contamination in the chemicals produced in each of eight plants in the study, knowledge of plant processes over time, and knowledge of what the job of each worker was across time. Each job in each plant was assigned an exposure score by the JEM. The exposure score represented a relative ranking of exposure for each worker. The rate ratios for all cancers (mortality) by septile of cumulative exposure score (15-year lag) were 1.00, 1.00, 1.29, 1.38, 1.43, 1.88, and 1.76 (*p*-value for trend < 0.001). Steenland et al. (1999) used data on exposure scores and serum level, which were available for 170 workers, to determine the relationship between exposure score and serum level. This enabled assignment of estimated serum level, based on the exposure score, for all workers (*n* = 3,538) in the study (Steenland et al. 2001). Analyses by septile of estimated cumulative serum level resulted in rate ratios for all cancers of 1.00, 1.26, 1.02, 1.43, 1.46, 1.82, and 1.62 (*p*-value for trend = 0.003).

Additional analyses of the Dutch cohort (Hooiveld et al. 1998) used a similar approach. Serum TCDD levels from 144 workers were used to build a model to predict serum levels based on duration of exposure, exposure during an accident, and exposure before 1970. Predicted serum TCDD values from the model were assigned to the whole cohort of 1,031 workers. Workers were then classified as having received low, medium, or high exposure based on predicted serum TCDD values. Workers who received medium and high exposures had significant 5-fold increases in cancer mortality compared with workers at the same plant with low dioxin exposure.

Becher et al. (1998) and Flesch-Janys et al. (1998) used a similar approach to further analyze a German cohort in detailed exposure–response analyses. TCDD levels from a sample of 275 workers were used to construct a model based on job, age, and body mass index. This model predicted TCDD values over time for all 1,189 members of the cohort. These authors then used these data to estimate time-dependent cumulative exposure to TCDD in the serum for each cohort member. Prior analyses had been restricted to a fixed estimate of serum TCDD at the end of exposure. Rate ratios for all-cancer mortality by categorized ppt-years of TCDD were 1.00, 1.12, 1.42, 1.77, 1.63, and 2.19 (*p*-value for trend = 0.03) (Becher et al. 1998).

Crump et al. (2003) conducted a meta-analysis of three of these cohorts (Flesch-Janys 1998; Ott and Zober 1996; Steenland et al. 1999) and found a positive and significant exposure–response trend for all cancers. Crump et al. (2003) also showed that the slope of the dose response was not dependent on the pattern of the risk in heavily exposed workers and that, by contrast, the slope was slightly steeper at lower doses.

Harking back to Austin Bradford Hill and his criteria for assessing causation (Hill 1965), positive exposure–response analyses are important in supporting the assessment of causality. Furthermore, the dose–response analyses are internal comparisons among workers and are unlikely to be affected by confounding from occupational, lifestyle, or other factors related to socioeconomic status. These positive exposure–response analyses for TCDD since the IARC classification (IARC 1997) strengthen the decision by IARC to label TCDD a human carcinogen.

### New results from Seveso.

Besides the new exposure–response findings, there has been new information from the Seveso cohort, which was exposed during an accident in Italy in 1976 (Bertazzi and di Domenico 2003). This cohort was exposed at one time to quite high levels of TCDD. People in zone A (the most highly exposed zone) had a median serum TCDD level of 72 ppt in 1992–1993 (back-extrapolated level to 1976, 379 ppt). In Seveso, exposure to nearly “pure” TCDD was well documented and affected all ages and both sexes. The exposed and reference populations both lived in a fairly homogeneous area and shared environmental, occupational, social, and cultural features. The limitations of the cohort are that the number of highly exposed subjects is relatively small and that follow-up has been of relatively short duration. However, recent data have shown significant cancer excesses that were not previously evident in this cohort.

Among those with relatively high exposure at Seveso (zones A and B), all-cancer mortality in the 20-year postaccident period and all-cancer incidence in the 15-year postaccident period failed to exhibit significant departures from the expected (Bertazzi et al. 2001; Pesatori et al. 2003). Among men, however, after 20 years of follow-up, both all-cancer (166 deaths) and lung cancer mortality (57 deaths) tended to be higher than expected [all cancer: relative risk (RR) = 1.1; 95% CI, 1.0–1.3; lung cancer: RR = 1.3; 95% CI, 1.0–1.7]. Furthermore, some specific cancer sites were significantly elevated. For lymphopoietic neoplasms, significant increases in mortality (20 years; RR = 1.7; 95% CI, 1.2–2.5) and morbidity (15-year latency; RR = 1.8; 95% CI, 1.2–2.6) were observed, consistent in both sexes. Furthermore, there was an increase in rectal cancer mortality in men (RR = 2.4; 95% CI, 1.2–4.6); a corresponding increase was seen for incidence. Among women, liver cancer incidence was elevated in the 15-year postaccident period (RR = 2.4; 95% CI, 1.1–5.1). Finally, in a separate analysis of 981 women in zone A who had stored serum, breast cancer incidence was significantly related to serum TCDD levels (a 2-fold increase for a 10-fold increase in serum TCDD), based on a limited number of cases (*n* = 15) (Warner et al. 2002).

### Other new studies.

Another cohort with well-documented exposure, based on serum TCDD levels, is the Ranch Hand cohort of Air Force personnel who sprayed Agent Orange in Vietnam. This cohort was not exposed to TCDD at the high levels of the industrial cohorts but nonetheless was exposed to levels considerably beyond background. For example, the mean serum TCDD level in the mid-1980s was 46 ppt (geometric mean, 15), compared with a mean of 233 ppt among the NIOSH cohort in the late 1980s (Fingerhut et al. 1991). Until recently, the Ranch Hand cohort had not shown any cancer excesses, and the number of cancers was small. Although there is still no overall cancer excess [standardized incidence ratio (SIR) = 1.07), in the most recent update (through 1999) of this cohort, Akhtar et al. (2004) found a significant excess of melanoma [(SIR = 2.57; 95% CI, 1.52–4.09) when comparing Ranch Hand personnel with the general population (16 cases)]. This excess did not appear among other Air Force personnel who were also in Southeast Asia in the 1960s but did not spray Agent Orange. Furthermore, there appeared to be an exposure–response trend, using several different measures of exposure. Akhtar et al. (2004) also found excesses of prostate cancer incidence, but these occurred in both exposed and nonexposed Air Force personnel and may have been due to increased cancer surveillance in both groups; both are subject to intense medical follow-up.

Other dioxin studies published since 1997 include a study of Army Chemical Corps veterans who did or did not serve in Vietnam (Dalager et al. 1997), and an update of a subcohort contained within the NIOSH cohort (Bodner et al. 2003). The studies are largely uninformative because the numbers are quite small or because exposure is uncertain (Dalager et al. 1997).

## Dioxin Risk Assessments

A separate issue is whether the findings that high levels of TCDD exposure lead to cancer has relevance for those exposed at low doses, that is, the general public. The classification of TCDD as a human carcinogen in 1997 strengthened the pressure to lower human exposure and was followed by subsequent World Health Organization (WHO) risk assessments that lowered considerably the accepted tolerable daily intake from previously set limits (WHO 1998 WHO 2001). There have also been several cancer risk assessment efforts to date (Becher et al. 1998; Crump et al. 2003; Starr 2001; Steenland et al. 2001; U.S. EPA 2000) using data on the high-exposure industrial cohorts to estimate risk at low doses. It should be noted that some of the high-exposure cohorts did have a fair number of low-exposed subjects, so the usual problem of extrapolating findings from high dose to low dose is not as pronounced as for some other agents for which risk assessment has been based on occupational cohorts. Nearly all these assessments concur in showing an appreciable excess risk of cancer due to relatively small increases above background levels. In the general population, such increases would be due to increased TCDD in the diet.

## New Evidence on the AhR

Apart from new epidemiologic data since 1997, there are also new experimental studies (some of them used in the recent WHO risk assessments) and advances in the understanding of mechanisms of action of dioxins, particularly concerning the AhR. The AhR is a nuclear receptor and transcription factor. In the presence of TCDD, it forms an active heterodimer with the aromatic hydrocarbon nuclear translocator (ARNT/HIF-1β) and induces (or suppresses) the transcription of numerous genes, including P4501A1 (*CYP1A1*) (Whitlock 1999). In the last few years, additional components of the AhR complex have been identified, including the AhR repressor, AhR-interacting protein (also known as XAP2), Rb protein, receptor-interacting protein 140, SRC-1, p23, and the RelA NF-κB subunit (Carlson and Perdew 2002; Kumar and Perdew 1999; Mimura et al. 1999; Petrulis and Perdew 2002). Molecular mechanisms occurring downstream of AhR and possibly associated with cancer development, such as changes in cytosolic signaling proteins, calcium mobilization, tumor suppressor proteins, growth factors, oncogenes, and cell cycle proteins, have been characterized (Carlson and Perdew 2002; Enan et al. 1998; Matsumura 2003).

Recently, molecular epidemiology investigations have been conducted on random samples of the Seveso population highly exposed to TCDD (zones A and B) and from the reference noncontaminated area (non-ABR) to evaluate how TCDD exposure affects the AhR pathway in human subjects *in vivo* (Baccarelli et al. 2004; Landi et al. 2003). Because of the extremely long biologic half-life of TCDD, plasma TCDD levels were still substantially elevated in the exposed subjects, particularly in females and older subjects (Landi et al. 1997). Experimental studies indicate that, after a transient increase, cellular levels of AhR decrease following TCDD binding (Pollenz 2002). Nearly 20 years after the Seveso accident, the levels of AhR transcripts (measured in uncultured peripheral blood lymphocytes) were decreased in the exposed subjects and negatively correlated with current plasma TCDD levels (Landi et al. 2003). These results show that TCDD exposure causes a persistent alteration of the AhR pathway in human subjects and are consistent with down-regulation of this receptor, comparable with that observed in several other receptor-mediated systems (Pollenz 2002). The impact on the health of exposed individuals of the persistent decrease of AhR transcripts, which in turn may affect any AhR-regulated biologic function, is to be clarified. Down-regulation tends to decrease the amount of receptor available for ligand binding and to attenuate the resulting biologic responses. Thus, the AhR, like most receptor systems, may have high initial sensitivity to the ligand, whereas in the presence of high amounts of TCDD, down-regulation would buffer against excessive ligand-induced responses. High initial levels of exposure, rather than low persisting exposures, may be associated with the highest effects. In the industrial cohorts, cumulative exposure predicts cancer excess. However, it is likely that cumulative and peak exposures are highly correlated among industrial workers. The new evidence from animal studies and on the AhR should be used to refine quantitative risk assessment of TCDD and could modify estimates on tolerable intake in humans. This evidence, put together, supports the approach taken by IARC to consider the animal and mechanistic data in the evaluation of carcinogenicity of these compounds in humans.

## Conclusion

The IARC classification of TCDD as a group 1 carcinogen (IARC 1997) has stirred some controversy. For example, Cole et al. (2003) argue that the original IARC classification of epidemiologic evidence for TCDD as “limited” (IARC 1997) was incorrect, claiming that “inadequate” would have been more appropriate (i.e., a causal interpretation was not “credible”). However, these authors ignored the original IARC focus on high-exposure subcohorts, ignored the positive exposure–response analyses, and raised the issue of possible confounding by smoking and other chemical carcinogens without any serious consideration of whether such possible confounding is likely, or whether it could account for the observed elevation of all-cancer mortality in those with higher TCDD exposure.

In our view, the epidemiologic and toxicologic evidence since the IARC (1997) classification of TCDD as a human carcinogen has strengthened the case for IARC’s decision. Furthermore, the dose–response assessments for TCDD and cancer indicate that TCDD exposure levels close to those in the general population may be carcinogenic and argue for caution in setting the upper ranges of long-term permissible exposure to dioxins.

## Figures and Tables

**Table 1:** Four industrial cohorts that served as a basis for IARC (1997) TCDD determination.

Study originally available to IARC in 1997*^a^*	Cancer SMR (95% CI) and definition of subcohort	No. of cancer deaths	Estimated TCDD at end of exposure*^b^*	Exposure–response data for TCDD
Fingerhut et al. 1991	1.5 (1.2–1.8), > 1 year exposure, 20 years of latency (59% of cohort)	114	Mean 418 ppt (*n* = 119)	Positive significant trend (*p* < 0.001, *p* = 0.003) in Steenland et al. (1999, 2001),*^c^* based on JEM and serum levels
Becher et al. 1996	1.3 (1.0–1.5), workers in two plants with documented chloracne and high serum TCDD levels	105	Plant 1: mean, 141 ppt (*n* = 190). Plant 2: mean, 402 ppt (*n* = 20)	Positive significant trend (*p* < 0.01) in Flesch-Janys et al. (1995), in Flesch-Janys et al. (1998; *p* = 0.01),*^c^* and in Becher et al. (1998; *p* = 0.03),*^c^* based on JEM and serum levels
Hooiveld et al. 1996	1.5 (1.3–1.9), workers in the most highly exposed plant (*n* = 549)	51	Geometric mean, 286 ppt (*n* = 48)	Medium- and high-exposure groups elevated (RRs = 4.7 and 4.1) versus low (Hooiveld et al. 1998),*^c^* based on work history and serum levels
Ott and Zober 1996	1.9 (1.1–3.0), chloracne and ≥20 years’ latency (*n* = 113)	18	Geometric mean, 400 ppt (*n* = 138)	Positive significant trend (*p* = 0.05) in original 1996 publication, based on body burden

Abbreviations: CI, confidence interval; NIOSH, National Institute for Occupational Safety and Health; SMR, standardized mortality ratio.

a IARC (1997; Table 38).

b IARC (1997; Table 22).

c Post-1997 findings.
